# Effects of Oral Rutaecarpine on the Pharmacokinetics of Intravenous Chlorzoxazone in Rats

**DOI:** 10.5487/TR.2008.24.3.195

**Published:** 2008-09-01

**Authors:** Sudeep R. Bista, Sang Kyu Lee, Dinesh Thapa, Mi Jeong Kang, Young Min Seo, Ju Hyun Kim, Dong Hyeon Kim, Yurngdong Jahng, Jung Ae Kim, Tae Cheon Jeong

**Affiliations:** 16 College of Pharmacy, Yeungnam University, 214-1, Dae-dong, Gyeongsan, 712-749 Korea; 26 Bioanalysis and Biotransformation Research Center, KIST, Seoul, 130-650 Korea

**Keywords:** Rutaecarpine, Chlorzoxazone, Interaction, Pharmacokinetics, CYP2E1, *In vivo*

## Abstract

It has been reported that hepatic microsomal cytochrome P450 (CYP) 2E1 is responsible for the metabolism of chlorzoxazone (CZX) to 6-hydroxychlorzoxazone. The present study was undertaken to assess the possible interaction of rutaecarpine, an alkaloid originally isolated from the unripe fruit of *Evodia rutaecarpa*, with CZX. Male Spraque-Dawley rats were administered with 80 mg/kg/day of oral rutaecarpine for three consecutive days. Twenty four hr after the pre-treatment with rutaecarpine, the rats were treated with 20 mg/kg of intravenous CZX Rat hepatic microsomes isolated from rutaecarpine-treated rats showed greater (50% increase) activity of p-nitrophenol hydroxylase (a marker of CYP2E1) when compared with the control rats. Compared with control rats, the AUC of CZX was significantly smaller (84% decrease) possibly due to significantly faster CL (646% increase) in rats pretreated with rutaecarpine. This could be, at least partially, due to induction of CYP2E1 by rutaecarpine.

## INTRODUCTION

Chlorzoxazone (CZX), once used as a skeletal muscle relaxant, is primarily metabolized to 6-hydroxychlorzoxazone (HCX) by hepatic microsomal cytochrome P450 (CYP) 2E1 in humans (Conney and Burns, 1960) and rats (Rockich and Blouin, 1999). HCX fromation has been used as a chemical probe to assess the activity of CYP2E1 *in vitro* and *in vivo* because of good correlation with CYP2E1 in humans (Peter *et al.*, 1990) and in rats (Rockich and Blouin, 1999).

Herbal remedies have become very popular as alternative medicines. Thus, the possible drug interaction with herbal medicines should be taken into consideration (Han *et al.*, 2007; Kim and Chun, 2007; Yang and Lee, 2007). The fruit of *Evodia rutaecarpa*, for example, has been used for the treatment of gastrointestinal disorders, headache and amenorrhea (Ueng *et al.*, 2002). Rutaecarpine (8,13-dihydro-7*H*-indolo-[2',3':3,4]-pyrido-[2,1-b]-quinazolin-5-one) is an alkaloid originally isolated from the unripe fruit of *Evodia rutaecarpa* (Chiou *et al.*, 1996). Rutaecarpine has recently been shown to have anti-inflammatory activity through cyclooxygenase-2 inhibition (Moon *et al.*, 1999; Woo *et al.*, 2001).

From a previous study, rutaecarpine is known to induce the CYP2E1 in mice based on measuring enzyme activity (Lee *et al.*, 2004). However, the studies on rats did not seem to have been reported, although it could be expected that the pharmacokinetic parameters of CYP2E1 substrates like CZX could be changed in rats with rutaecarpine. The purpose of this paper was to report changes in the pharmacokinetics of intravenous CZX with oral rutaecarpine pretreatment in rats. In this study, the p-nitrophenol hydroxylase activity (CYP2E1 marker) was also measured in rat liver microsomes following rutaecarpine pretreatment.

## MATERIALS AND METHODS

**Chemicals.** Rutaecarpine (Fig. 1; purity, > 99%) was synthesized by our groups (Lee *et al.*, 2001). CZX, β-glucuronidase (Type H-3; from *Helixpomatia*), p-nitrophenol and the reduced form of β-nicotinamide adenine dinucleotide phosphate (β-NADPH; as a trisodium

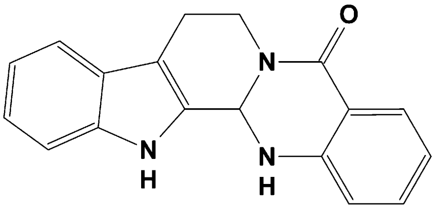

**Fig. 1.** A chemical structure of rutaecarpine.

salt) were purchased from Sigma Chemical Co. (St. Louis, MO, USA). 3-Aminophenyl sulfone, an internal standard for the high-performance liquid chromatographic (HPLC) analysis of CZX, was a product from Gentest Corporation (Woburn, MA, USA). Other chemicals were of analytical grade or HPLC grade.

**Animals.** Specific pathogen-free male Sprague-Dawley rats (5~6 weeks old and weighing 250~280 g) were purchased from the Orient Company (Seoul, South Korea). The rats were acclimated for at least one week. Upon arrival, rats were housed ($n = 3$ per cage) in strictly controlled rooms of 23 ± 3°C, 12-hr light (07:00~19:00) and dark cycles and 50 ± 10% relative humidity with the intensity of 150~300 Lux. All rat experiments were performed based on a guideline recommended by the Society of Toxicology (USA) in 1989.

**Intravenous study.** Rats were randomly divided into two groups, without (control; $n = 3$) and with ($n = 3$) oral rutaecarpine. Rats received oral rutaecarpine dissolved in corn oil at a dose of 80 mg/kg/10 ml/day using a stomach tube once a day for consecutive three days, followed by an injection of 20 mg/kg CZX (1 ml/kg dissolved in 1% povidone solution) via penile vein one day after the last dose of rutaecarpine. The same volume of corn oil was administered into the control rats. A blood sample (300 μl) was collected via the subclavian vein immediately before (control) and 0.04, 0.08, 0.17, 0.33, 0.67, 1, 1.5, 2, 3, 4 and 6 hr after the injection of CZX. The blood sample was centrifuged (3,000 *g*, 15 min) at 4°C to obtain serum sample. Other procedures used were similar to a reported method (Frye and Stiff, 1996).

**Preparation of liver microsomes.** The same dose of rutaecarpine was orally administered to rats for consecutive three days. The control group received the same volume of corn oil. Twenty four hr after the last dosing, all rats were subjected to necropsy. The liver was perfused with ice-cold saline via hepatic portal vein. Then, the livers were removed and homogenized with four volumes of ice-cold 0.1 M potassium phosphate buffer (pH, 7.4). The liver microsomes were isolated by the differential centrifugation and stored at -80°C until use (Lee *et al.*, 2004a). The content of hepatic microsomal protein was determined using bovine serum albumin (Lowry *et al.*, 1951).

**Measurement of p-nitrophenol hydroxylase (PNPH) activity.** PNPH activity was determined as described by Koop (1986). The reaction mixture (1.0 ml) included 1 mM NADPH in 0.1 M potassium phosphate buffer (pH 7.4) containing 100 μM p-nitrophenol and liver microsomes of rats without (control; $n = 3$) or with ($n = 3$) pretreatment with rutaecarpine. After thorough mixing, the mixture was incubated for 30 min in a water-bath shaker (37°C, 100~105 rpm). After centrifugation at 25°C (3000 *g*, 10 min), a 100 μl of 10 N NaOH was added to 1 ml supernatant fraction. The amount of 4-nitrocatechol formed was determined using UV-Visible spectrophotometer (UV-1061, Shimadzu, Kyoto, Japan) at 512 nm.

**HPLC analysis of CZX.** The concentrations of CZX in the above biological samples were analyzed by the reported HPLC method (Frye and Stiff, 1996) with slight modifications. Briefly, a 100 μl of 0.1 M potassium phosphate buffer (pH, 7.4) containing 200 units of β-glucuronidase was added to a 100 μl serum samples. HCX is reportedly excreted in the urine as a glucuronide conjugate (Conney *et al.*, 1960; Desiraju *et al.*, 1983). For this reason, β-glucuronidase was incubated with serum samples to hydrolyze the glucuronidated metabolites in the present study. After thorough mixing and incubation in a water-bath shaker (55°C, 100~105 rpm) for 1 h, a 10 μl of 43% phosphoric acid, a 100 μl of methanol containing 40 μg/ml of 3-aminophenyl sulfone (internal standard), and 1 ml ethyl acetate were added. After mixing, the samples were centrifuged (3,000 *g*, 15 min) at 15°C. The upper organic layer (0.85 ml) was transferred to a clean tube and evaporated in a water-bath under a gentle stream of nitrogen gas at 37°C. The residue was reconstituted with 0.1 ml of methanol and centrifuged (9,000 *g*, 15 min) at 15°C. Then, a 20 μl was directly injected onto the HPLC column [Inertsil® ODS-3 column; particle size, 5 μm; 4.6 mm, i.d. × 150 mm, *l.*; GL Sciences Inc., Tokyo, Japan). The mobile phase, acetonitrile:20 mM ammonium formate buffer (pH, 6.8) (65:35, v/v) was run at a flow rate of 1.0 ml/min, and the column eluent was monitored using an UV detector at 287 nm. The retention time and detection limit of CZX were 8.1 min and 0.01 μg/ml, respectively. The coefficient variations were less than 15%.

**Pharmacokinetic analysis.** The total area under the plasma concentration-time curve from time zero to time infinity (AUC) was calculated using the trapezoidal rule-extrapolation method (Chiou, 1978). The area from the last datum point to time infinity was estimated by dividing the last measured plasma concentration by the terminal-phase rate constant.

Standard methods (Gibaldi and Perrier, 1982) were used to calculate the following pharmacokinetic parameters using a non-compartmental analysis (WinNonlin; version 2.1; Scientific Consulting Inc., Cary, NC, USA); area under the curve to the blood concentration (AUC), terminal half-life, the time-averaged total body clearance (CL), the mean residence time (MRT), and the apparent volume of distribution at a steady state ($V_{ss}$).

**Statistical analysis.** A *p*-value < 0.01 was deemed to be statistically significant using an unpaired Student's *t*-test. All results were expressed as the mean ± standard error of the mean (S.E.M.).

## RESULTS AND DISCUSSION

Many drugs were originally derived from herbs and other natural resources. In many regions of the world, herbal medicines have been used for treating diseases and disorders for thousands of years. Many herbal preparations are claimed to be effective in treating diseases, but in most cases the active ingredient(s) in these herbal mixtures are unknown and the mechanisms of action are obscure. For this reason, many researchers enthusiastically work in the field of the identification of new constituents with biological activities, the total or semi-synthesis of new constituents, and characterization of their metabolic profiles *in vitro* and *in vivo*. Rutaecarpine is an excellent example of a biologically active constituent from natural resources, not only because it could induce some CYP enzymes, but also because its metabolic pathway including phase 1 and phase 2 has clearly been characterized (Lee *et al.,* 2004a, b, 2005, 2006).

From the reports of the inductive effects of rutaecarpine on drug-metabolizing enzymes, it would be expected that repeated use of rutaecarpine-containing herbal medication could cause interactions with some drugs. However, the possible drug-drug interaction has hardly been investigated. To date, studies concerning the interaction of rutaecarpine with theophylline (Jan *et al.*, 2005; Ueng *et al.*, 2005) and caffeine (Tsai *et al.*, 2005) have been performed. Recently, the pharmacokinetic parameters of acetaminophen that could be affected by rutaecarpine in rats have been reported

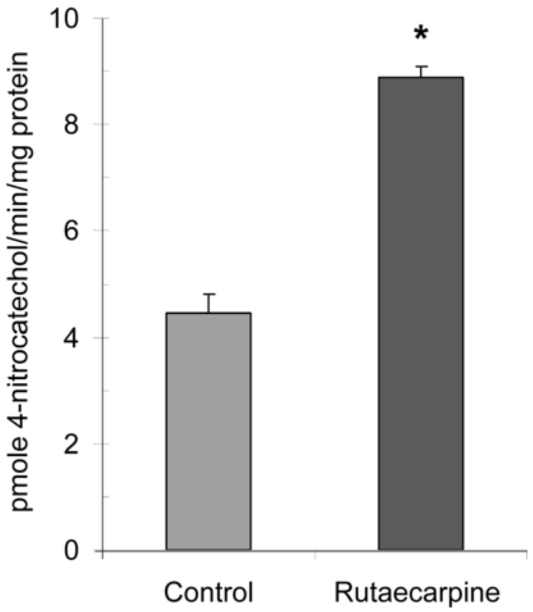

**Fig. 2.** Effect of rutaecarpine on the formation of 4-nitrocatechol (p-nitrophenol hydroxylase activity) in rat liver microsomes (*n* = 3, each). Each value represents the mean ± S.E.M. *Significantly different (*p* < 0.01) from the control.

(Lee *et al.*, 2007). To demonstrate the drug-drug interaction or herb-drug interaction of rutaecarpine with CZX, we have determined the *in vivo* pharmacokinetic parameters of CZX, following pre-treatment with rutaecarpine in the present study.

Fig. 2 shows the PNPH activity in rat hepatic microsomes without (control) and with pretreatment with

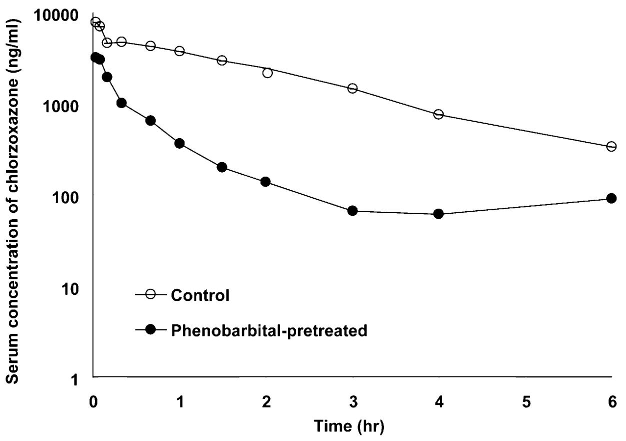

**Fig. 3.** Mean serum concentration-time profiles of intravenous CZX in rats without (○; *n* = 3) or with (●; *n* = 3) rutaecarpine. Rats were pre-treated with 80 mg/kg oral rutaecarpine for 3 days, and administered 20 mg/kg intravenous CZX one day after the last dose of rutaecarpine. Each value represents mean.

**Table 1.** Pharmacokinetic parameters of CZX in rats without (control) and with rutaecarpine pretreatment

| Parameter | Control ($n$ = 3) | With rutaecarpine ($n$ = 3) |
|---|---|---|
| AUC (μg·hr/ml) | 12.49 ± 1.44 | 1.98 ± 0.32* |
| Terminal half-life (min) | 1.43 ± 0.10 | 2.17 ± 0.60 |
| CL (ml/min/kg) | 0.41 ± 0.05 | 2.65 ± 0.43* |
| MRT (min) | 2.04 ± 0.15 | 2.32 ± 0.38 |
| $V_{ss}$ (ml/kg) | 0.85 ± 0.15 | 6.00 ± 0.99* |

*Significantly different ($p < 0.01$) from the control.

rutaecarpine. The results showed a significant difference in the formation of 4-nitrocatechol between two groups of rats. The rutaecarpine-treated rats showed almost 50.1% increase in the formation of 4-nitrocatechol than control rats, indicating that rutaecarpine could induce CYP2E1 responsible for the metabolism of CZX. Thus, it could be expected that the pharmacokinetics of CZX are changed by rutaecarpine.

Fig. 3 shows the mean serum concentration-time curves of CZX for two groups of rats, and the relevant pharmacokinetic parameters of CZX are summarized in Table 1. Compared with control rats, rutaecarpine pretreatment caused a significantly smaller AUC of CZX (84.1% decrease), and this could have been due to significantly faster CL (646% increase) (Table 1). The faster CL was, at least in part, attributable to induction of CYP2E1 by rutaecarpine (Fig. 2). In addition, the MRT and half-life of CZX in rats with rutaecarpine pretreatment were longer than those in control rats, although these were not significant. Meanwhile, the present study should be extended to the time point showing steady state level of serum CZX to understand the reason why the serum level of CZX was declined so rapidly.

In summary, the present results indicated that rutaecarpine could act as an inducer of hepatic CYP2E1 enzyme, which plays a major role in the biotransformation of chemicals such as CZX. Hence, the administration of CZX following rutaecarpine pre-treatment causes pharmacokinetic alterations in the metabolism of CZX in rats.

## ACKNOWLEDGEMENTS

This work was supported by the grant from Korea Research Foundation (KRF-2006-E00154).
